# Liver Damage Associated with *Polygonum multiflorum* Thunb.: A Systematic Review of Case Reports and Case Series

**DOI:** 10.1155/2015/459749

**Published:** 2015-01-12

**Authors:** Xiang Lei, Jing Chen, Jingtian Ren, Yan Li, Jingbo Zhai, Wei Mu, Li Zhang, Wenke Zheng, Guihua Tian, Hongcai Shang

**Affiliations:** ^1^Tianjin University of Traditional Chinese Medicine, 88 Yuquan Road, Tianjin 300193, China; ^2^Center for Drug Reevaluation, State Food and Drug Administration, Xicheng, Beijing 100045, China; ^3^Second Affiliated Hospital of Tianjin University of Traditional Chinese Medicine, 816 Zhenli Road, Tianjin 300150, China; ^4^Dongzhimen Hospital, Beijing University of Chinese Medicine, Dongcheng, Beijing 100007, China; ^5^Key Laboratory of Chinese Internal Medicine of Ministry of Education and Beijing, Dongzhimen Hospital, Beijing University of Chinese Medicine, Beijing 100700, China

## Abstract

*Objective*. To summarize the characteristics and analysis of relevant factors and to give references for prevention and further study of liver damage associated with *Polygonum multiflorum* Thunb. (HSW), we provide a systematic review of case reports and case series about liver damage associated with HSW. *Methods*. An extensive search of 6 medical databases was performed up to June 2014. Case reports and case series involving liver damage associated with HSW were included. *Results*. This review covers a total of 450 cases in 76 articles. HSW types included raw and processed HSW decoction pieces and many Chinese patent medicines that contain HSW. Symptoms of liver damage occur mostly a month or so after taking the medicine, mainly including jaundice, fatigue, anorexia, and yellow or tawny urine. Of the 450 patients, two cases who received liver transplantation and seven who died, the remaining 441 cases recovered or had liver function improvement after discontinuing HSW products and conservative care. *Conclusion*. HSW causes liver toxicity and may cause liver damage in different degrees and even lead to death; most of them are much related to long-term and overdose of drugs. Liver damage associated with HSW is reversible, and, after active treatment, the majority can be cured. People should be alert to liver damage when taking HSW preparations.

## 1. Introduction

**Figure 1:**
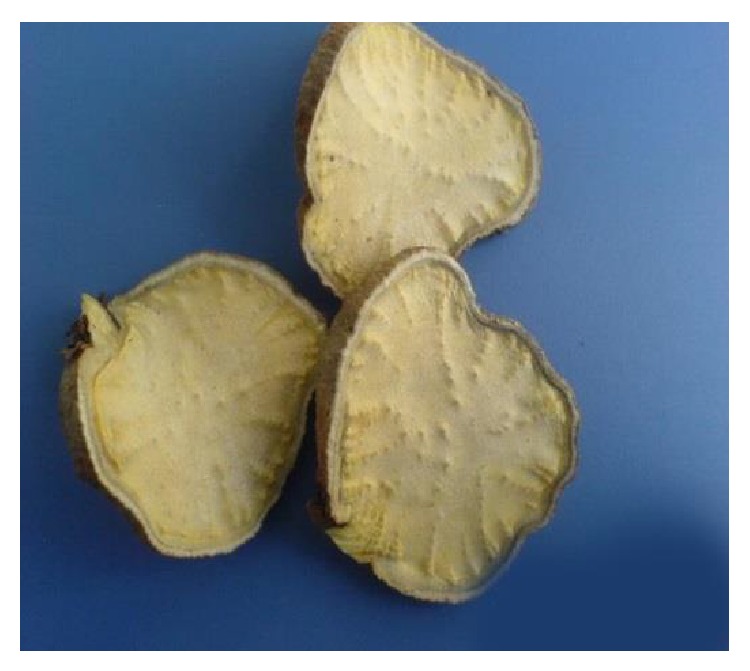
Raw HSW.

**Figure 2:**
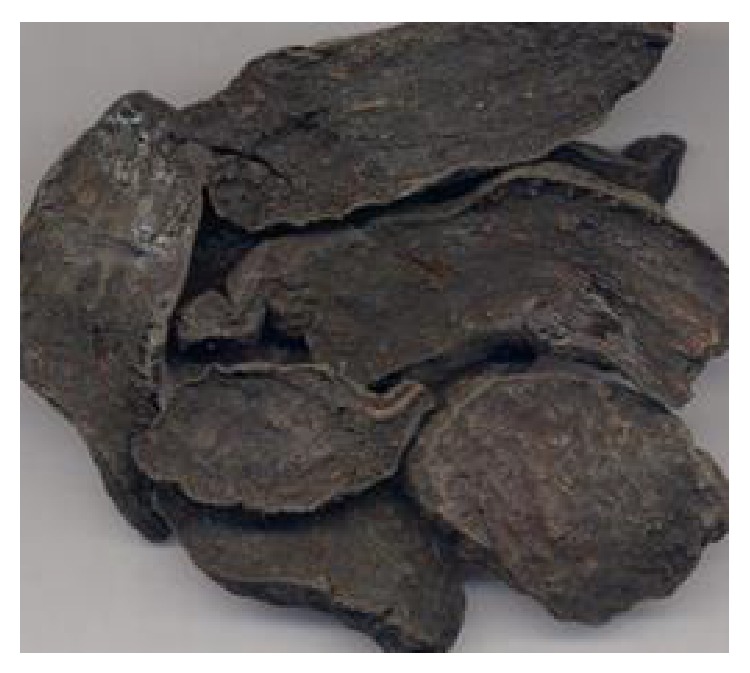
Processed HSW.

*Polygonum multiflorum* Thunb. (He Shou Wu in Chinese pinyin, hereinafter referred to as HSW) is the root of* Polygonum multiflorum*, a member of Polygonaceae. As a Chinese herb, it was recorded most early in “Kaibao Bencao” published by the imperial court of the Song Dynasty (973-974 A.D.) [1]. In the* Chinese Pharmacopoeia* (2010), there are two forms of HSW decoction pieces: raw state (natural root) (Figure 1) and processed form, that is, radix polygoni multiflori preparata (boiled in black-bean liquid according to a traditional process) (Figure 2). The two forms have different properties: the raw HSW is used for detoxification, eliminating carbuncle, preventing malaria, and relaxing bowel, whereas the processed HSW is used for nourishing liver and kidney, supplementing essence and blood, blackening hair, strengthening bones and muscles, eliminating dampness, and reducing lipid [2]. Modern pharmacological studies have shown that HSW have the effect of reducing blood lipid and antiarteriosclerosis [3–5], protecting liver [6, 7], enhancing immunologic [8, 9], improving memory, protecting nerve cells, and increasing intelligence [10, 11], antioxidation, antiaging [12, 13], and so forth.

HSW is popular in many countries, especially in China. Traditional Chinese herbal medicine ordinarily recommends the use of herbs in complex formulas, but HSW is also often taken as a single herb. While there are many HSW-containing products, the most well-known product is like Shou-Wu-Pian, which is usually consumed as an antiaging product, or as a tonic for dizziness with tinnitus, and also appears to be efficacious in the treatment of premature greying of hair, lumbago, spermatorrhea, leucorrhea, and constipation. With the wide application, liver damage associated with HSW has been reported in China, Korea, Japan, Britain, Italy, Australia, and other countries [14, 15]. To fully understand the characteristics and possible factors of hepatitis associated with HSW, we conduct a comprehensive review of the relevant published literatures; we hope that our findings can provide guidance for clinical medication and scientific research and thus can help to avoid hepatitis induced by HSW in the future.

## 2. Methods 

### 2.1. Data Sources and Search Strategy

In June 2014, we searched the following Chinese-language electronic databases: Chinese Biomedical Literature Database (CBM, 1980–2014), Chinese Journal Full-Text Database (CNKI, 1980–2014), Weipu Journal Database (VIP, 1989–2014), and Wanfang Data (1990–2014) and two English-language databases PubMed and EMBASE (1989–2014). The search terms included* Polygonum multiflorum*, radix polygoni multiflori, He Shou Wu, liver injury, liver damage, liver diseases, hepatitis, and liver failure in English or Chinese. These terms were searched as free-text in the title or the abstract. The references of relevant reviews and the included literatures were checked for possible identification of additional studies.

### 2.2. Study Selection

Case reports and case series are included in the review if they reported factual data on liver damage related to HSW. Clinical trials, reviews, commentaries, and other nonrelated literatures were excluded. Some clinical trials of HSW that reported liver damage were excluded for reasons originally given by Lao et al., specifically that they were too small to provide convincing evidence of rare complications [16]. Two authors (Xiang Lei and Jing Chen) independently examined the titles and abstracts of all papers found through the search to determine if they fulfilled the inclusion criteria outlined above. The full texts of potentially relevant articles were retrieved for detailed assessment. Differences can be resolved through discussion.

### 2.3. Data Extraction

After screening, articles were read in full and extracted by two independent reviewers (Xiang Lei and Jing Chen). Information on author, year of publication, number of patients, disease originally treated, gender, dose and dosage forms, course of taking HSW, clinical manifestations, length of hospitalization, outcome and family history, and recurrence of hepatitis related to HSW were extracted according to the designed data extraction table independently by two authors.

## 3. Results

### 3.1. Search Process and Result

The search identified 923 database records, which led to the retrieval of documentation on 103 studies for a full-text review. One additional reference was identified while searching reference lists of the included papers. 76 articles (59 case reports and 17 case series) met the inclusion criteria. In total, 450 cases of hepatitis associated with HSW were reported. The characteristics of the case reports and case series are shown in Tables 1 and 2. Because case series is summary report for multiple patients and because of the lack of description of the individual patient information, the following is mainly an analysis of case reports.

### 3.2. Gender and Age

In 450 cases, 224 (49.78%) were male and 226 (50.22%) were female. In 72 cases of case reports, patients ranged in age from 5 to 78 years, with a median of 36.5. The most commonly affected age group was 18–44 (37/72, 51.4%), followed by 45–59 (24/72, 33.3%), <18 (6/72, 8.3%), and ⩾60 (5/72, 6.9%).

### 3.3. Reasons for Medication (Disease Originally Treated)

In case series, the causes of consuming HSW products include grey hair, hair loss, hypertension, coronary heart disease, hyperlipidemia, osteoarthritis, insomnia, dizziness, and health care; the top diseases were gray hair and hair loss [77–91]. The reasons for medication of 72 cases of case reports are shown in Table 3.

### 3.4. Dosage Forms and Usage

In case series, there is insufficient information on HSW forms and usage of each patient, but it is worth noting that, in two case series, the ratio of processed HSW to raw HSW is 5 : 13 and 25 : 11, respectively [79, 76]. The HSW forms of case reports are shown in Table 4; in terms of usage, all involved Chinese patent medicines are of oral formulation; the types of HSW decoction pieces used included tea, liquor, powder, and decoction.

### 3.5. Dose and Duration of Taking HSW

In case series, there is insufficient information on dose and duration of taking HSW of each patient, but some articles indicate that the majority of patients take Chinese patent medicine in accordance with the instructions. For the 28 cases who consumed Chinese patent medicines in case reports, 17 (17/28, 60.7%) cases are in accordance with the recommended dosage and the remaining are unknown. Of the 44 cases who used HSW decoction pieces, 19 cases (19/44, 43.2%) did not report the daily dose; the other 25 cases' daily dose ranged from 1 to 100 g with a median of 15 g; when the daily dose was ⩽12 g (11/25), the median of duration is 30 d, but if the daily dose is >12 g (14/25), the median of duration is 60 d. For all the patients of case reports, the duration from starting to take HSW to the onset of symptoms ranged from 1 to 240 days, with a median of 30 days (Table 5).

### 3.6. Clinical Manifestations and Outcome

According to case reports and case series, most of the patients had been hospitalized with jaundice, fatigue, anorexia, and yellow or tawny urine. A handful of patients were found with abdominal distension, abdominal pain, diarrhea, rash, pruritus, and other symptoms. After admission examination, a few cases were found with epigastrium tenderness, first percussion over the liver, hepatomegaly or splenomegaly, and even ascites [77, 86, 89]. Nine case series reported liver damage types of 221 patients, including 132 (132/221, 59.7%) cases of hepatocytes type, 34 (34/221, 15.4%) cases of cholestatic type, and 55 (55/221, 24.9%) cases of mixed type.

In addition to two (2/450, 0.4%) cases who received liver transplantation [78, 85] and seven (7/450, 1.6%) who died [79, 89, 85–82], the remaining 441 (441/450, 98%) cases recovered or had liver function improvement after discontinuing HSW products and conservative care. In case reports, the length of 64 patients' hospitalization ranged from 6 to 120 d, with a median of 29.5 (Table 6). The case series that reported the average length of hospitalization was about four weeks [79, 91, 89, 81–92].

### 3.7. Recurrence and Family History

*In case reports, *23 (23/72, 31.9%) cases were reported with liver damage associated with HSW for many times, and 3 (3/72, 4.2%) cases had family history of HSW induced hepatitis [61–71]. In case series, seven articles reported 53 (53/138, 38.4%) cases with liver damage related to HSW many times [77, 91, 82, 83–92].

## 4. Discussion 

Based on the above information we know that HSW associated with liver injury can occur at any age group and with no gender orientation. The main reason for using HSW is that patients suffer from gray hair and hair loss; this may be associated with those patients who are more likely to use HSW products, but whether patients suffering from gray hair or hair loss are prone to occurrence of liver damage associated with HSW remains to be further studied.

Although many studies suggest that processing could reduce the toxicity of HSW [15, 93], all HSW products may lead to liver damage regardless of herbal processing. In the* Chinese Pharmacopoeia *(2010), predetermined daily dose of raw HSW is 3–6 g and of processed HSW is 6–12 g [2]. Although only 25 cases of case reports have the HSW dosage information, 14 cases exceed maximum dosages (12 g/d). The results show that, when daily dose is less than 12 g, from the beginning of consuming HSW to the occurrence of liver damage, the median time is 60 days, while when daily dose is more than 12 g the median time is 30 days; this suggested that HSW associated with liver damage has a “dose-time-toxicity” relationship; animal experiments also had proved it [93].

Because more than 30% of patients in case reports and cases series were reported liver damage occurred many times which was induced by HSW, and a few cases have a family history of liver damage induced by HSW; these suggested that HSW associated with liver damage may be related to personal body factors of patients.

The mechanism of HSW induced liver damage is still unclear and mainly toxic substances are also an uncertainty [15, 94]; even few animal experiments did not find hepatotoxicity of HSW [95, 96]. Some studies suggested that the adverse reactions of traditional Chinese medicine are closely related to patient self-medication, arbitrarily increase in the dose, or long-term use [97–99]; this systematic review and some papers included in it also have the same standpoint.

HSW can cause different degrees of liver injury, even need of liver transplantation (2/450), and death (6/450). However, most HSW induced liver injuries are reversible; after withdrawal of HSW products and corresponding treatments, the vast majority of patients can recover liver function.

Because HSW induced liver injury is not a specific diagnostic method and some cases included in this review also take other drugs or herbal medicines, so considering the doctors experience, medical technology, and other limiting factors may not show all liver injury is caused by HSW.

## 5. Conclusion

Many cases of liver damage associated with HSW had been reported worldwide; HSW has liver toxicity and may cause different degrees of liver damage. The liver damage in most patients is reversible, after discontinuation of HSW products, and active treatment can restore liver function, but there are also a small number of patients with liver failure and even death. We suggest that patients should take HSW products under the guidance of a physician or pharmacist and avoid using them for long-term or in high-dose. If fatigue, anorexia, nausea, yellowing of skin and sclera, yellow urine, and other symptoms appear after medication, patients should be alerted to the occurrence of liver damage and promptly stop the medicine and treatment.

## Figures and Tables

**Table 1:** Patient details recorded from published case reports.

Reference	Age/gender	Reasons for medication	HSW type	Administration and dosage	Duration of intake, day	Hospitalization, day	Outcome	Rechallenge?
Liu et al. [17]	20/F	Grey hair	HSW	OAP, 15 g	42	10	Recovery	NA
Miao and Yun [18]	52/F	Health care	P. HSW	OAP, 5 g	20	14	Recovery	NA
	61/F	Hyperlipidemia	R. HSW	SIW, 5 g	180	12	Recovery	NA
Zhao [19]	39/M	Grey hair	Jing-Wu-Pian	Oral, 3 × 6 tablets	60	18	Recovery	NA
Tan [20]	48/F	NA	P. HSW	uNA, 10 g	40	30	Recovery	NA
Liu et al. [21]	37/M	Vitiligo	P. HSW	OAP, 10 g	30	35	Recovery	NA
Gao et al. [22]	14/F	Vitiligo	P. HSW	Decoction, NA	38	15	Recovery	NA
Baňárová et al. [23]	33/F	NA	SWW	Oral, NA	60	NA	Recovery	NA
Gao [24]	45/M	Hair loss	P. HSW	OAP, NA	90	21	Recovery	NA
Kui and Chen [25]	43/F	Grey hair	HSW	OAP, 8–10 g	60	30	Recovery	NA
Shao and Li [26]	50/M	Grey hair	HSW	OAP, NA	60	30	Recovery	NA
	40/F	Health care	R. HSW	Decoction, 10–20 g	60	50	Recovery	NA
Hu et al. [27]	36/M	Chronic nephritis	P. HSW	OAP, 15 g	30	37	Recovery	NA
Zhen and Zeng [28]	52/F	Grey hair	P. HSW	OAP, NA	14	30	Recovery	NA
Li and Zhang [29]	50/F	Grey hair	R. HSW	OAP, 2 g	60	15	Recovery	NA
	28/F	Hair loss	R. HSW	OAP, 3 g	75	12	Recovery	NA
Chen et al. [30]	57/M	Grey hair	R. HSW	NA	1	20	Recovery	NA
Liu et al. [31]	56/F	Grey hair	Yishen wufa	Oral, 2 × 10 mL	120	21	Recovery	NA
Sun [32]	39/F	Allergic rhinitis	R. HSW	Decoction, 15–20 g	30	28	Recovery	NA
	72/F	Health care	R. HSW	Decoction, 10–20 g	60	42	Recovery	NA
Cao et al. [33]	28/M	Grey hair	P. HSW	NA, 10 pieces	90	42	Recovery	NA
Liu et al. [34]	26/M	Hair loss	Yangxue shengfa	Oral, 2 × 4 capsules	60	30	Recovery	NA
Yan [35]	28/M	Hair loss	HSW	Decoction, 4 g	90	28	Recovery	NA
Wu and Niu [36]	47/F	Hair loss	Yishen wufa	Oral, 3 × 10 ml	20	14	Recovery	NA
Liu et al. [37]	50/F	Hair loss	SWW	Oral, NA	7	25	Recovery	NA
Chen et al. [38]	46/F	Health care	HSW tea	Oral, 1-2 g	60	25	Recovery	NA
	57/M	Health care	HSW tea	Oral, 1-2 g	20	15	Recovery	NA
Yun et al. [39]	51/F	Grey hair	SWW	Oral, 2 × 6 g	180	18	Recovery	NA
Furukawa et al. [40]	53/F	NA	SWP	NA	240	60	Recovery	NA
Yan et al. [41]	54/F	Grey hair	HSW	OAP, 2 × 10 g	60	35	Recovery	NA
He [42]	31/F	Constipation	HSW	Oral, NA	15	10	Recovery	NA
Chen [43]	38/F	Grey hair	HSW	OAP, NA	120	63	Recovery	NA
Cho et al. [44]	34/M	NA	HSW	HSW tea (liquor), NA	30 (4)	34	improved	NA
Wang et al. [45]	34/M	Health care	R. HSW	SIW, NA	20	20	Recovery	NA
	46/M	Health care	R. HSW	SIW, NA	10	27	Recovery	NA
	49/F	Health care	R. HSW	SIW, NA	5	27	Recovery	NA
Zhu [46]	36/M	Hair loss	R. HSW	SIW, NA	30	30	Recovery	NA
Yang and Li [47]	38/M	Grey hair	HSW	Decoction, 30 g	Once a week (3w)	NA	Recovery	NA
	51/M	Sequelae of stroke	HSW	Decoction, 15 g	15	NA	Recovery	NA
Laird et al. [48]	35/M	Thinning hair	NuHair	Oral, NA	NA	120	Recovery	NA
Yang and Dong [49]	54/M	Hair loss	SWP	Oral, 3 × 6 tablets	40	17	Recovery	NA
	26/M	Grey hair	SWP	Oral, 3 × 6 tablets	30	59	Recovery	Yes
Fu and Yu [50]	32/F	Infertility	P. HSW	Decoction, NA	18	Untreated	Recovery	NA
Zhao [51]	17/M	Grey hair	SWW	Oral, 3 × 6 tablets	40	90	Recovery	Yes
Li [52]	65/M	Dizziness, tinnitus	HSW	SIW, 30–50 ml	20	30	Recovery	Yes
	38/M	Grey hair	SWP	Oral, 3 × 6 tablets	30	20	Recovery	NA
Cárdenas et al. [53]	28/F	Hair loss	Shen-Min	Oral, 2 tablets	56	6	Recovery	NA
Han [54]	42/F	Grey hair	HSW	OAP, 10 g	90	30	Recovery	Yes
Yang [55]	35/M	Grey hair	P. HSW	Decoction, 30 g	45	50	Recovery	NA
Panis et al. [56]	5/F	NA	SWP	Oral, 3 tablets	120	30	Recovery	Yes
Chen et al. [57]	20/F	Grey hair	HSW	OAP, NA	20	40	Recovery	NA
Huo and Ling [58]	28/M	Hair loss	SWP + Yangxue shengfa	Oral, 3 × 5 tablets and 2 × 4 capsules	60	18	Recovery	Yes
Mazzanti et al. [59]	78/M	Chronic prostatitis	SWP	Oral, RD	30	NA	Recovery	NA
Shao [60]	46/F	Hair loss	R. HSW	Decoction, NA	6	30	Recovery	Yes
	45/M	Grey hair, hair loss	R. HSW	Decoction, 100 g	NA	30	Recovery	Yes
Leng [61]	15/F	Grey hair	HSW	Decoction, 15 g	30	NA	Recovery	NA
Dai and Li [62]	18/F	Grey hair	SWP	Oral, NA	90	90	Recovery	NA
Yuan [63]	66/F	Constipation	HSW ointment	Oral, NA	7	30	Recovery	Yes
Sun [64]	20/F	Neurasthenia	SWP + Liuwei Dihuang Wan	Oral, 3 × 6 tablets and 2 × 9 g	3	10	Recovery	Yes
Yang [65]	17/M	Grey hair	SWP	Oral, NA	14	29	Recovery	Yes
Park et al. [66]	46/F	Grey hair	SWP	Oral, RD	14	30	Recovery	NA
Sheng [67]	38/F	Grey hair	HSW	Oral, NA	6	NA	Recovery	Yes
Li et al. [68]	58/F	Dizziness	HSW	Decoction, 30 g	7	15	Recovery	Yes
	49/M	Hypertension	HSW	SIW, 15 g	10	20	Recovery	Yes
Fan and Zhou [69]	26/M	Grey hair	SWP	Oral, 3 × 6 tablets	30	59	Recovery	Yes
Li [70]	17/M	Grey hair	SWP	Oral, 3 × 5 tablets	20	60	Recovery	Yes
Ye [71]	30/M	Grey hair	HSW	Decoction, 50 g	2	94	Recovery	Yes
Zhang [72]	36/F	NA	SWP	Oral, 10 ml	15	15	Recovery	Yes
Niu [73]	28/M	Grey hair	SWP	Oral, 3 × 5 tablets	10	60	Recovery	Yes
He and Zhen [74]	19/F	Grey hair	SWP	Oral, NA	NA	30	Recovery	Yes
	27/F	Grey hair	SWP	Oral, NA	NA	NA	Recovery	Yes
But et al. [75]	31, F	Dizziness	SWP	Oral, RD	Several weeks	21	Recovery	Yes

SWP: Shou-Wu-Pian; SWW: Shou-Wu-Wan; OAP: oral administration of powder; SIW: soaked in water to drink; P. HSW: processed HSW; R. HSW: raw HSW; HSW: unclear whether it was processed; RD: recommended dosages; NA: not available.

**Table 2:** Characteristics of included case series.

Reference	Number of cases	Gender (F/M)	Age (a)	Duration of intake, day	Type of liver injury (number)	Outcome
Dong et al. [76]	18	5/13	18–63	1–120	H (18)	18 recoveries
Lian et al. [77]	52	22/30	22–69	5–120	H (30); C (9); M (13)	52 recoveries
Zhang et al. [78]	13	11/2	35–66	4–15	H (6); C (4); M (3)	12 recoveries; 1 LT
Zhang et al. [79]	36	13/23	24–73	NA	H (21); C (2); M (13)	33 recoveries; 1 cirrhosis; 2 deaths
Ding [80]	65	45/20	34–71	7–90	NA	64 recoveries; 1 death
Guo [81]	15	8/7	18–57	7–56	H (8); C (3); M (4)	15 recoveries
Xie et al. [82]	10	3/7	46.1 ± 10.2	30–105	H (5); C (4); M (1);	9 recoveries; 1 death
Song [83]	26	12/14	38–71	9–93	NA	26 recoveries
Wang [84]	20	7/13	34–67	NA	NA	20 recoveries
Jung et al. [85]	25	7/18	24–65	1–180	H (18); M (7)	23 recoveries; 1 LT; 1 death
Chen et al. [86]	12	5/7	20–70	15–90	H (4); C (4); M (4)	12 recoveries
Liu and Li [87]	7	5/2	31–64	60–180	NA	7 recoveries
Liu [88]	9	3/6	34–68	NA	NA	9 recoveries
Xu et al. [89]	40	24/16	45.2	9–168	H (22); C (8); M (10)	38 recoveries; 2 deaths
Yang et al. [90]	13	8/5	32–68	7–30	NA	13 recoveries
Zhou and Qiu [91]	11	5/6	34–58	NA	NA	11 recoveries
Zhang et al. [92]	6	3/3	24–50	4–24 w	NA	6 recoveries

NA: not available; H: hepatocellular; C: cholestatic; M: mixed; LT: liver transplantation.

**Table 3:** Use reasons of case reports.

Disease originally treated	Patients (number)
Gray hair, hair loss	43 (59.7%)
Health care	8 (11.1%)
Dizziness	3 (4.2%)
Vitiligo	2 (2.8%)
Constipation	2 (2.8%)
Others^a^	8 (11.1%)
Unknown	6 (8.3%)

Total	72 (100%)

^a^Including infertility, hypertension, allergic rhinitis, hyperlipidemia, cerebral infarction sequelae, chronic nephritis and neurasthenia, and chronic prostatitis.

**Table 4:** The classification of the drug of case reports.

Dosage forms	Patients (number)
Chinese patent medicine	
Shou-Wu-Pian	17 (23.61%)
Shou-Wu-Wan	4 (5.56%)
Yishen wufa	2 (2.78%)
Others^b^	5 (6.94%)
Decoction pieces	
R. HSW	13 (18.06%)
P. HSW	10 (13.89%)
Unknown	21 (29.17%)

Total	72 (100%)

^b^Including Huolisu Koufuye, Jing-Wu-Pian, NuHair, Shen-Min, and Yangxue shengfa capsules.

**Table 5:** The duration of taking HSW of case reports.

Duration (day)	Patients (number)
<10	10 (13.89%)
10–30	27 (37.50%)
31–60	19 (26.39%)
>60	12 (16.67%)
Unknown	4 (5.56%)

Total	72 (100%)

**Table 6:** The length of hospitalization of case reports.

Hospitalization (d)	Patients (number)
<15	8 (11.1%)
15–30	37 (51.4%)
31–60	14 (19.4%)
>60	5 (6.9%)
Unknown	8 (11.1%)

Total	72 (100%)
